# Safe usage of cosmetics in Bangladesh: a quality perspective based on microbiological attributes

**DOI:** 10.1186/s40709-015-0033-4

**Published:** 2015-09-09

**Authors:** Rashed Noor, Nagma Zerin, Kamal Kanta Das, Luthfun Naher Nitu

**Affiliations:** Department of Microbiology, Stamford University Bangladesh, 51 Siddeswari Road, Dhaka, 1217 Bangladesh; Department of Chemical Engineering, University of Waterloo, 200 University Avenue West, Waterloo, ON N2L3G1 Canada

**Keywords:** Cosmetics, Microbiological quality, User safety, Public health

## Abstract

The present review attempted to emphasize on the microbiological quality of the commonly used cosmetics item by the majority of the Bangladeshi community. The abundance of contaminating microorganisms has been quantitatively discussed and the possible health risk has been focused upon usage of these items. Only a very few research efforts have been conducted on the cosmetic items in Bangladesh so far. The microbiological contamination aspects have been portrayed in this review using the information collected from a substantial number of cosmetic items which were earlier subjected to extensive microbiological and biochemical analyses. The prevalence of bacteria, fungi and the specific pathogenic microorganisms has been discussed based on research so far locally conducted on the finished items sold in markets, especially within the Dhaka metropolis. The laboratory scale experiments revealed the presence of enormous number of bacteria, actinomycetes and fungi within the commonly used cosmetics. Conversely, the anti-bacterial activity was noticed in some of the products which might be in favor of the user safety. The prevalence of pathogenic microorganisms in the cosmetic items certainly raises a substantial public health issue. The necessity of the routine microbiological testing of the commonly used cosmetic items as well as the legislative measures to mitigate the contamination problem is thus of great significance.

**Electronic supplementary material:**

The online version of this article (doi:10.1186/s40709-015-0033-4) contains supplementary material, which is available to authorized users.

## Background

Cosmetic items have long been used by the people around the world in order to enhance personal appearance and maintain personal hygiene and safety [1–5]. Varieties of cosmetics with discrete functions are currently available (they are being used for the care of each part of body) [6–8]. Thus, the cosmetic items are the principal categories of health care products besides the pharmaceutical products which are mainly used for mitigation of diseases [6–8]. The cosmetic items are in general known as the chemical substances or sometimes the preparations from natural herbs which are frequently applied to human body exclusively with an intention of beautification as well as for cleaning and protection from various hazards [1, 2, 9, 10]. Besides the chemical reactivity or the side effects of cosmetic usage, another aspect of discussion relies on the microbiological quality of the cosmetic products [2, 11]. Though the cosmetic items are considered principally as the health care products, since they are largely under the non-sterile pharmaceutical categories, the items may undergo microbial contamination [2, 12–14]. The extent of microbial contamination largely depends on the microorganism-infected bulk ingredients during product manufacturing and processing accompanied with insufficient in-process quality examinations followed by unfussy storage condition as well as the distribution into the markets without apposite quality assurance of the finished cosmetic products [2, 10, 15, 16]. Such contamination in the cosmetics items may result in several diseases including scabies, acne, eczema, dyschromia and other skin diseases [8, 17–21].

**Fig. 1:**
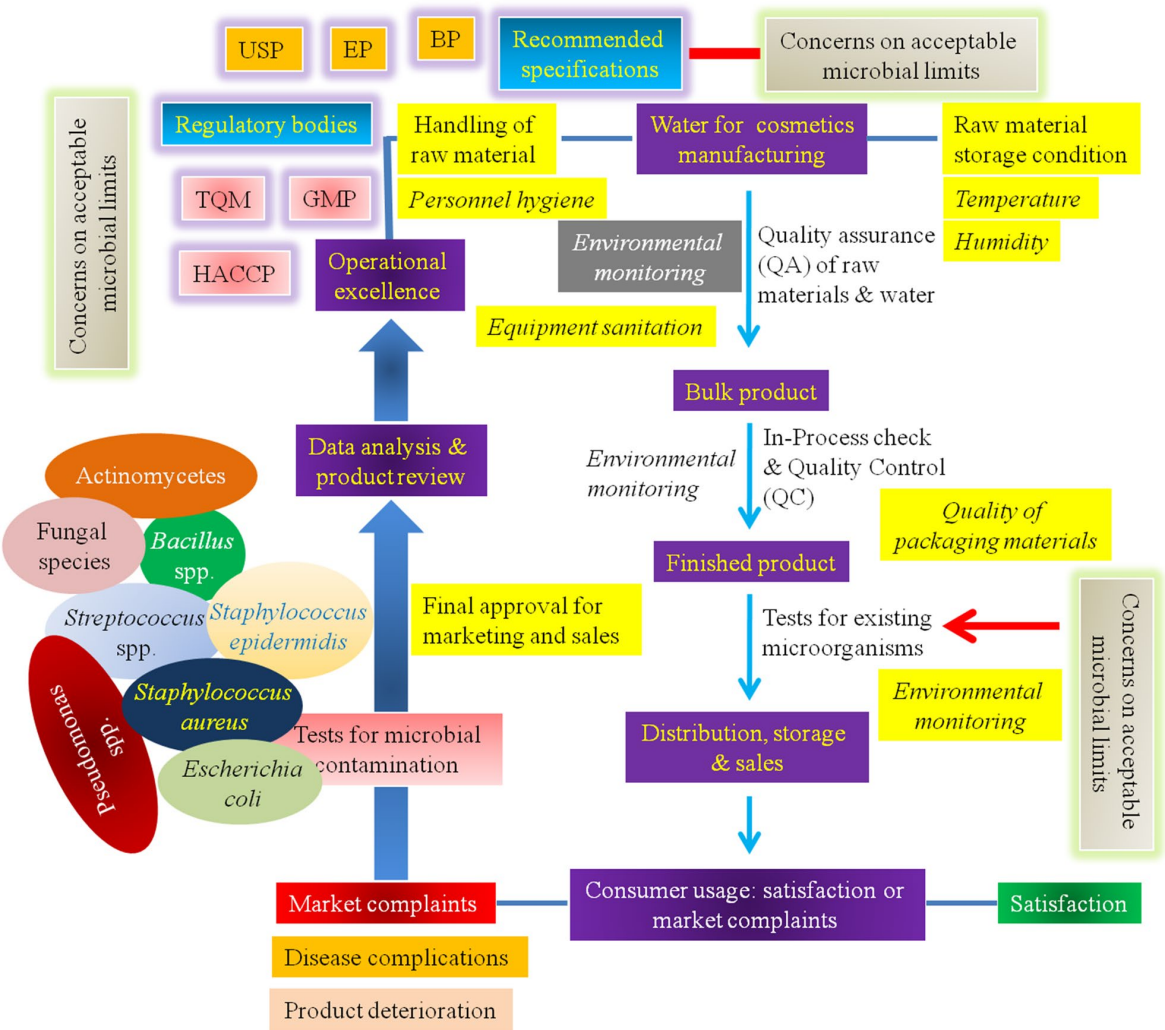
Regulatory scheme for maintenance of the microbiological quality of cosmetic products. The figure illustrates the possible microbiological entry into the cosmetics products. The microbial load should be examined at every stage of manufacturing and packaging till the finished product formation, and should be intermittently checked during storage and distribution. The bio-burden is required to meet the specification criteria as recommended by the British Pharmacopeia (BP), European Pharmacopeia (EP) or the United States Pharmacopeia (USP). Market complaints need to be handled carefully to ensure the further product quality employing the good manufacturing practice (GMP) and total quality management (TQM). The critical points of microbial access into the products are required to be monitored by means of the “hazard analysis: critical control point” (HACCP) implementation [51]

Like the pharmaceutical products, cosmetics are quite likely to consist of various substrates as the product ingredients which in turn may unfortunately further support the growth of a range of pathogenic bacteria and fungi [3, 9]. Therefore, these health care products need to be free from pathogens to reduce the possibility of the impairment of skin and mucous membranes [3, 17]. The incidence of skin diseases is likely to be frequent in the developing countries due to the unhygienic environment, dense population favoring contagious diseases, lack of awareness on cleanliness, improper sanitation practices, and finally the massive use of contaminated processing water [22–26]. Hence this is imperative that a proper maintenance of sound microbiological quality of the cosmetic products’ manufacturing starting from raw materials as well as the manufacturing waters according to the guidelines stated in the Good Manufacturing Practice (GMP) and the Food and Drug Administration (FDA) is stringently required (Fig. 1) [27–31]. Indeed, the clear understanding the requirement of microbiological analyses of raw materials and final products of cosmetics for obtaining products with good microbiological quality in accordance to the microbial limit recommended in the British Pharmacopeia (BP), United States Pharmacopeia (USP) or the European Pharmacopeia (EP) and other regulatory bodies is thus essential, too [30–34]. Pharmaceutical and cosmetic industries in Bangladesh have been expanding for the last two decades with a great scope of maintenance of public health safety and the associated business as well [35]. Unlike the pharmaceutical medicaments, the foremost health concern relies on the lack of cosmetic testing aptitudes as well as the inadequate facilities in the poor settings, like in Bangladesh [2]. Since a large number of users are dependent on the various cosmetic items, it is of essence to visualize the extent of microbial contamination within these products as has been rationalized above. Along this line, the present review discusses the frequency and type of microbiological contamination of the commonly used cosmetic products in Bangladesh and focuses on the prevalence of health deteriorating pathogenic bacteria in terms of public health issues.

## Review

### Microorganisms associated with the cosmetic items: global and Bangladeshi perspectives

Contamination of cosmetic products by an array of pathogenic bacteria like *Staphylococcus aureus*, *Pseudomonas aeruginosa*, *Streptococcus* spp., *Micrococcus* spp., *Clostridium tetani*, *Bacillus cereus*, actinomycetes and fungi has been reported worldwide; however, as stated earlier, such knowledge is scarce in Bangladesh [10, 13, 14]. Until recently, Bangladeshi scientific community remains quite reluctant about cosmetic microbiology; very interestingly, microbiological contamination aspects were resolved almost 30 years ago. In 1946, the first notification of microbial contamination was made in talcum powder by *Clostridium tetani*, and then in 1967, *Klebsiella pneumoniae* was reported to contaminate hand creams, and finally in 1983, aqueous soaps were observed to harbor *Pseudomonas stutzeri* [30]. The principal reasons behind such contamination has been chalked out afterwards, and the microbiological experiments demonstrated that the unhygienic handling of bulk ingredients during manufacturing followed by the insufficient in-process check principally account for such contamination [2, 36–39]. However, certain acceptable limits of microorganisms within the cosmetic items have been recommended by the FDA and BP/USP. For example, in case of cosmetic items to be applied within the non-eye area, the Total Aerobic Microbial Count (TAMC) should be no more than 10^3^ cfu g^−1^; and for the items used within the eye area, the limit should not exceed 10^2^ cfu g^−1^ [1, 8, 18]. The microbial burden over the recommended limit may result in several types of diseases as described above [7–20].

Microbial access into the production stream of cosmetic manufacturing as in pharmaceuticals is very likely to occur with a nearly similar mechanism [40]. Common microorganisms gaining access into the products and premises may include several Gram-negative and Gram-positive bacteria and the opportunistic pathogens [32]. Such contaminants may possess the potential of adapting to the ingredients within the products and may survive [41]. Microbial contaminants may be sourced from the poor quality raw ingredients, manufacturing equipments, processing environment and personnel, even within the packaging materials [12, 32, 42, 43]. Indeed, the microbial contamination of the products kept in market for sales purpose is also possible even after all in-process checks during manufacturing and packaging, possibly due to the (1) microbiological entry into the active ingredients or the additives (i.e., preservatives) which remained un-noticed; (2) due to the improper handling and discrepancy in the storage conditions of the products (Fig. 1).

In Bangladesh, the microbiological contamination of the pharmaceutical medicaments has widely been reported locally so far [44–48]. However, reports on the cosmetic products quality are not that much available like the pharmaceutical ones [2, 49–51]. Tests for finished cosmetic products have been conducted principally by a group of researchers employing the recommended microbiological and biochemical tests [31, 44–54]. Varieties of soaps, shampoo, lotions, face washes, creams and petroleum jelly, whereby almost all samples were found to be hugely contaminated with bacteria and fungi (Additional file 1). The TAMC was noticed to be within a range of 10^3^–10^5^ cfu g^−1^ whereas the Total Yeast and Mould Count (TYMC) was observed up to 10^3^ cfu g^−1^. Samples were found to harbor several pathogenic bacteria; i.e., *Staphylococcus* spp., *Pseudomonas* spp. and *Bacillus* spp. within a range of 10^1^–10^3^ cfu g^−1^. Presence of *Klebsiella* spp. was also noted up to 10^1^ cfu g^−1^. In a recent study, among 10 categories of samples (total 30 items), almost all samples were found to possess the TAMC and TYMC up to 10^5^ cfu g^−1^ and 10^3^ cfu g^−1^, respectively [49]. Growth of *Staphylococcus* spp., *Pseudomonas* spp. and *Klebsiella* spp. was also noted. Taken together, the microbiological survey on a total of 50 items of cosmetic products revealed the microbial load in an unsafe level which further leads to greater public health risk associated principally with skin diseases among the users. For maintaining the sustainable quality of the cosmetic products, as for pharmaceuticals, the widely implemented concept of Hazard Analysis of Critical Control Points (HACCP) can be employed to improve the microbiological safety of the finished products [31, 32, 55]. Interestingly, with an opposite trait to microbiological proliferation within the cosmetic items, the anti-bacterial activity was observed in seven samples against *Staphylococcus* spp., *E. coli*, *Bacillus* spp., *Pseudomonas* spp., *Klebsiella* spp. and *Listeria* spp. employing the methods described earlier [56–58]. Besides the microbiological contamination aspect of the cosmetics products, such anti-bacterial trait of these items may, in contrary, draw an overall public health impact of the cosmetic samples tested. Overall, the microbiological investigation on the commonly used cosmetic revealed a huge microbial contamination exceeding the recommended limits. Stern actions on the microbiological quality control along with the personal hygienic improvement during cosmetic products processing would be effective for the enhanced management of the mass public health.

### Recommendations

Although the microbial analysis of the cosmetic products described above has been done in small scale, still the sample size of 50 was quite enough to indicate valid results on the current microbiological status of the products in context to project microbiological management of cosmetic products in Bangladesh. However, routine microbiological tests of all cosmetic items sold in market are required, and the necessary steps should be taken by Bangladeshi Government, too. Besides the regular tests of the finished products by the cosmetic manufacturing industries, additional quality tests of the market items can be initiated by the universities and research organizations in Bangladesh, thereby raising the firmness of quality of the sold products among the users.

Another vital problem is that in many cases cosmetic oriented skin irritations that are noticed within Bangladeshi people remain undiagnosed [59]. Appropriate diagnosis of the diseases caused by cosmetic is definitely required. Furthermore, as many drug oriented diseases are also emerging within the Bangladeshi community mostly due to the microbial drug-resistance traits as well as the microbial contamination of finished products, appropriate microbiological analyses are required not only for disease diagnosis or the pharmaceutical product quality, but also for the sound formation of the finished forms of the cosmetic products [51]. Besides, rigorous measures need to be taken by the Bangladeshi legislative bodies to ensure the practice and commencement of GMP maintenance in the cosmetic manufacturing industries. The operational sites must comply with the FDA requirements and appropriate quality assurance (QA) system must be endorsed during the market survey market complaints.

The manufacturing and packaging processes of the cosmetic items should be strictly maintained by specifications given by the Federal Food, Drug and Cosmetic Act (FD and C Act) and the Fair Packaging and Labeling Act (FPLA) [60]. Regular inspection of cosmetic manufacturing facilities is required to assure cosmetic product safety and to determine whether cosmetics are adulterated or misbranded under the FD & C Act or FPLA. According to the International Standard Organization (ISO) 22716 and FDA regulations, building premises, constructions and facilities used for manufacturing of cosmetic products should have adequate space together with built-in outfit to prevent cross contamination between raw materials, intermediate formulations, bulk materials and finished products. Periodic control of pests, sufficient lighting and ventilation, filtering of dust particles, maintenance of pharmaceutically ambient humidity and temperature, and frequent sanitary safeguarding in the manufacturing areas are worth to implement. All equipments should be maintained in clean conditions with regular calibration, and should be of defined design, size, and material in order to prevent particle droplet and microbial adhesion [61]. Thorough cleaning of containers and the hygiene of the workers along the production line should be accomplished according to the international and/or European laws and legislations. Together with regular microbiological tests (microbial limit tests, sterility tests, microscopy, etc.), the raw materials should be stored in closed containers and handled carefully to prevent mix-ups or selection errors, contamination with microorganisms or other chemicals, and degradation from exposure to excessive heat, cold, sunlight, moisture, etc. [61]. The entire system for the manufacturing water should be routinely monitored for the presence of pyrogens, and should be free from the possible development of biofilm. Another point is to ponder over the use of color additives in cosmetic items. When used in cosmetics, they must comply with the identity, specifications, uses, restrictions, and labeling requirements stated in the FD and C Act [62].

People in the cosmetic stores responsible for maintenance and sales of the finished products should be aware of keeping the storage conditions at the required level (for instance, at appropriate temperatures and humidity). The regulatory bodies both within the Governmental and the private sectors should take appropriate measures in coordination with the microbiology researchers, pharmacy professionals and the physicians around the country. Overall, as a number of diseases are emerging in Bangladesh, the appropriate handling of cosmetic products would definitely aid in reducing the ongoing common diseases [51, 63, 64].

## Conclusion

Pharmaceutical industries in Bangladesh are abundant and a number of pharmaceutical medicaments are available while some are imported from foreign pharmaceutical manufacturers. In contrast, cosmetic manufacturing industries in Bangladesh are very few, and as a result, plenty of cosmetic items are imported in Bangladesh. The laboratory based microbiological analyses of the pharmaceutical medicaments are in plenty in Bangladesh; however, such in vitro experiments on the cosmetic items are in scarce. Present review portrayed the informative description on the common cosmetic items in Bangladesh along with their microbiological status in accordance with the recommended limit by the international regulatory bodies. The laboratory investigations in small scale as described in this article unraveled that a major portion of cosmetic products might be contaminated with pathogenic microorganisms. Such public health concern should be addressed stringently, not only in Bangladesh perspective, but also within the other developing countries consisting of people mostly unconscious about the negative impact of the usage of cosmetics.

## Additional file

**Additional file 1.** Prevalence of pathogenic microorganisms in different types of cosmetics (cfu g^−1^).

## References

[1] Onurdağ FK, Özgen S, Abbasoğlu D (2010). Microbiological investigation of used cosmetic samples. Hacettepe Uni J Faculty Pharm.

[2] Das KK, Fatema KK, Nur IT, Noor R (2013). Prevalence of microorganisms in commonly used cosmetic samples in Dhaka Metropolis. J Pharma Sci Inno..

[3] Siegert W (2012). Microbiological quality management for the production of cosmetics and detergents. SOFW J..

[4] Gad GFM, Aly RAI, Ashour MSE (2011). Microbial evaluation of some non-sterile pharmaceutical preparations commonly used in the Egyptian market. Trop J Pharm Res..

[5] Perry B (2011). Cosmetic microbiology. Microbiol Today..

[6] Dashen MM, Chollom PF, Okechalu JN, Ma’aji JA. Microbiological quality assessment of some brands of cosmetics powders sold within Jos Metropolis, Plateau State. J Microbiol Biotech Res. 2011;1:101–6.

[7] Ravita TD, Tanner RS, Ahearn DG, Arms EL, Crockett PW (2009). Post-consumer use efficacies of preservatives in personal care and topical drug products: relationships to preservative category. J Ind Microbiol Biotechnol.

[8] Behravan J, Bazzaz F, Malaekeh P (2005). Survey of bacteriological contamination of cosmetic creams in Iran (200). Int J Dermatol.

[9] Herrera AG (2004). Microbiological analysis of cosmetics. Methods Mol Biol.

[10] Jimenez L, Ignar R, Smalls S, Grech P, Hamilton J, Bosko Y, English D (1999). Molecular detection of bacterial indicators in cosmetic/pharmaceuticals and raw materials. J Ind Microbiol Biot..

[11] Özalp M (1998). Microbiological contamination of cosmetic products. Turkey Clin J Cosmet..

[12] Baird RM, Bloomfield SFL (1996). Microbial quality assurance of cosmetics, toiletries and non-sterile pharmaceuticals.

[13] Elaine B (1989). The hazards of cosmetics.

[14] Smart R, Spooner DF (1972). Microbiological spoilage in pharmaceuticals and cosmetics. J Soc Cosmet Chem.

[15] SCCS. The SCCS’S notes of guidance for the testing of cosmetic substances and their safety evaluation. 8th Revision; 2012. http://ec.europa.eu/health/scientific_committees/consumer_safety/docs/sccs_s_006.pdf.

[16] Campana R, Scesa C, Patrone V, Vittoria E, Baffone W (2006). Microbiological study of cosmetic products during their use by consumers: health risk and efficacy of preservative systems. Lett Appl Microbiol.

[17] Mahé A, Ly F, Aymard G, Dangou JM (2003). Skin diseases associated with the cosmetic use of bleaching products in women from Dakar. Senegal. British J Dermatol..

[18] Pollack M, Mandal DL, Bennett JE, Dolin R (2000). *Pseudomonas aeruginosa*. Principles and practice of infectious diseases.

[19] Becks VE, Lorenzoni N (1995). *Pseudomonas aeruginosa* outbreak in a neonatal intensive care unit: a possible link to contaminated hand lotion. Am J Infect Control.

[20] Brannan DK, Dille JC (1990). Type of closure prevents microbial contamination of cosmetic during consumer use. Appl Environ Microbiol.

[21] Parker MT (1972). The clinical significance of the presence of micro-organisms in pharmaceutical and cosmetic preparations. J Soc Cosmet Chem.

[22] Prüss-Üstün A, Corvalán C (2006). Preventing disease through healthy environments: Towards an estimate of the environmental burden of disease.

[23] Cundell AM. Environmental monitoring in non-sterile product manufacturing. In: Moldenhauser J, editor. Environment monitoring. Davis Horwood/PDA; 2005. pp. 217–30.

[24] Cundell AM (2005). Managing the microbiological quality of pharmaceutical excipients. PDA J Pharma Sci Technol..

[25] Denyer SP, Hodges NA, Gorman SP, Hugo W, Russell A (2004). Pharmaceutical microbiology.

[26] United States Pharmacopeia (2003). (USP). Microbiological examination of non-sterile products: tests for specified microorganisms. Pharm. Forum..

[27] European Pharmacopoeia (EP), 4th edition. Council of Europe, Strasbourg; 2002.

[28] British Pharmacopoeia (BP). Her Majesty’s Stationery Office (HMSO). London, United Kingdom; 2003.

[29] British Pharmacopoeia (BP). The Stationery Office. London, United Kingdom; 2005.

[30] Baird RM, Baird RM, Hodges NA, Denyer SP (2000). Sampling: principles and practice. Handbook of microbiological quality control: pharmaceuticals and medical devices.

[31] Baird RM, Baird RM, Hodges NA, Denyer SP (2000). Culture media used in pharmaceutical microbiology. Handbook of microbiological quality control: pharmaceuticals and medical devices.

[32] Hugo WB, Russel AD (1998). Pharmaceutical Microbiology.

[33] Jahnke M (1997). Use of the HACCP concept for the risk analysis of pharmaceutical manufacturing processes. Eur J Parenter Sci..

[34] Lund W (1994). The pharmaceutical codex.

[35] Shaown SA (2011). Pharmaceutical Industry of Bangladesh.

[36] Khasru SM (2007). SME sector in Bangladesh: a critical overview. The Cost and Management.

[37] Mwambete KD, Justin-Temu M, Fazleabbas FS (2009). Microbiological assessment of commercially available quinine syrup and water for injections in Dar Es Salaam. Tanzania. Trop J Pharm Res..

[38] Kallings LO, Ringertz O, Silverstolpe L, Ernerfeldt F (1966). Microbial contamination of medical preparation. Acta Pharm Suec..

[39] Manu-Tawiah W, Brescia BA, Montgomery ER (2001). Setting threshold limits for the significance of objectionable microorganisms in oral pharmaceutical products. PDA J Pharm Sci Technol.

[40] Smith, JE. Biotechnology principles. In: Reinhold VN. Aspects of microbiology. Wokingham, Berkshire, England; 1985.

[41] Hodges NA, Baird RM, Hodges NA, Denyer SP (2000). Pharmacopoeial methods for the detection of specified micro-organisms. Handbook of microbiological quality control: pharmaceuticals and medical devices.

[42] Denyer SP, Baird RM (2007). Guide to microbiological control in pharmaceuticals and medical devices.

[43] Parker MS, Aulton ME (2000). Microbiological contamination and preservation of pharmaceutical preparations. Pharmaceutics: The science of dosage from design.

[44] Hossain MA, Raton KA, Noor R (2014). Microbiological quality investigation of eye and ear ointments available in Bangladesh. J Pharmacog Phytochem..

[45] Fatema K, Chakraborty SR, Sultana T, Rahman MM, Kamali NM, Das KK, Noor R (2014). Assessment of microbiological quality of the pediatric oral liquid drugs. J Pharmacog Phytochem..

[46] Rana J, Sultana T, Das KK, Noor R (2014). Microbiological analysis of topicals available in Bangladesh. Int J Pharm Pharma Sci..

[47] Raton KA, Hossain MA, Acharjee M, Noor R (2013). Assessment of microbiological quality and the anti-bacterial traits of sterile liquids used for medication of eye and ear infections in Bangladesh. Am J Pharma Health Res..

[48] Khanom S, Das KK, Banik S, Noor R (2013). Microbiological analysis of liquid oral drugs available in Bangladesh. Int J Pharm Pharmac Sci..

[49] Akon T, Das KK, Nitu LN, Noor R (2015). Demonstration of in vitro anti-bacterial activity of the popular cosmetics items used by the Dhaka locality. Asian Pac J Trop Dis..

[50] Hossain SMJ (2009). Importance of the bioburden test in pharmaceutical quality control. Pharma Microbiol Forum Newsletters..

[51] Noor R, Zerin N, Das KK (2015). Microbiological quality of pharmaceutical products in Bangladesh: current research perspective. Asian Pac J Trop Dis..

[52] Jawetz E (1987). Review on medicinal microbiology.

[53] Lundov MD, Moesby L, Zachariae C, Johansen JD (2009). Contamination versus preservation of cosmetics: a review on legislation, usage, infections, and contact allergy. Contact Dermatitis..

[54] Cappuccino JG, Sherman N (1996). Microbiology - A laboratory manual.

[55] Denyer SP, Baird RM (1990). Guide to microbiological control in pharmaceuticals.

[56] Jagessar RC, Mars A, Gones G (2008). Selective antimicrobial properties of leaf extract against various micro-organisms using disc diffusion and agar well diffusion method. J Nat Sci..

[57] Clinical and Laboratory Standards Institute (CLSI). Methods for dilution antimicrobial susceptibility tests for bacteria that grow aerobically: Approved standard-seventh edition. Wayne, Pennsylvania, USA: CLSI; 2006.

[58] Carson CF, Hammer KA, Riley TV (1995). Broth micro-dilution method for determining the susceptibility of *Escherichia coli* and *Staphylococcus aureus* to the essential oil of *Melaleuca alternifolia* (tea tree oil). Microbios.

[59] Adhikary AB, Kamal S, Saha SK, Hossain A, Quader SA (2009). Badruzzaman, Chanda PK. Cosmetic approach of atrial septal defect repair through right anterolateral thoracotomy. Bangladesh Med Res Counc Bull.

[60] FDA Authority over Cosmetics (2014) Retrieved August 14, 2015 from http://www.fda.gov/Cosmetics/GuidanceRegulation/LawsRegulations/ucm074162.htm#What_kinds.

[61] Draft Guidance for Industry: Cosmetic Good Manufacturing Practices (2013). Retrieved August 14, 2015 from http://www.fda.gov/downloads/Cosmetics/GuidanceComplianceRegulatoryInformation/GuidanceDocuments/UCM358287.pdf.

[62] Color Additives Permitted for Use in Cosmetics (2014). Retrieved August 14, 2015 from http://www.fda.gov/Cosmetics/Labeling/IngredientNames/ucm109084.htm.

[63] Noor R, Munna MS (2015). Emerging diseases in Bangladesh: current microbiological research perspective. Tzu Chi Med J..

[64] Chowdhury FFK, Acharjee M, Noor R (2015). Maintenance of environmental sustainability through microbiological study of pharmaceutical solid wastes. Clean – Soil, Air. Water.

