# Supplementary material for: Safe usage of cosmetics in Bangladesh: a quality perspective based on microbiological attributes
Source: J Biol Res (Thessalon). 2015 Sep 9;22(1):10. doi: 10.1186/s40709-015-0033-4 (PMC4565015; doi:10.1186/s40709-015-0033-4)
Supplement: Supplementary file 1 — Additional file 1. Prevalence of pathogenic microorganisms in different types of cosmetics (cfu g−1). [file 40709_2015_33_MOESM1_ESM.doc]

**Additional file 1**

**Prevalence of pathogenic microorganisms in different types of cosmetics (cfu g^-1^)**

| Sample | TVB  (cfu g^-1^) | Total fungal count | *E. coli*  (cfu g^-1^) | *Klebshialla* spp. (cfu g^-1^) | *Staphylococcus*  spp. (cfu g^-1^) | *Pseudomonas*  spp. (cfu g^-1^) | *Actinomycetes* | *Bacillus*  spp. (cfu g^-1^) |
| --- | --- | --- | --- | --- | --- | --- | --- | --- |
| **Powder**  (Akon *et al.*, 2015) |  |  |  |  |  |  |  |  |
| Magic prickly-heat | 2.0×10^4^ | 6.0×10^4^ | 0 | 0 | 2.0×10^2^ | 2.0×10^1^ | 0 | ND |
| MarilTelcom powder | 3.0×10^5^ | 5.0×10^4^ | 0 | 0 | 3.0×10^2^ | 1.0×10^2^ | 0 | ND |
| Vatiny face powder | 2.1×10^4^ | 4.0×10^3^ | 0 | 0 | 1.4×10^3^ | 3.0×10^2^ | 0 | ND |
| **Deo Roll on**  (Akon *et al*., 2015) |  |  |  |  |  |  |  |  |
| Fa | 1.1×10^4^ | 4.0×10^3^ | 0 | 0 | 7.0×10^2^ | 2.0×10^2^ | 0 | ND |
| She | 3.0×10^4^ | 2.0×10^4^ | 0 | 0 | 3.0×10^3^ | 1.0×10^2^ | 0 | ND |
| Rexona men | 3.0×10^5^ | 2.2×10^4^ | 0 | 0 | 5.0×10^2^ | 2.0×10^2^ | 0 | ND |
| **Lipstics**  (Akon *et al*., 2015) |  |  |  |  |  |  |  |  |
| La femme | 5.0×10^5^ | 2.0×10^4^ | 0 | 0 | 2.0×10^3^ | 0 | 0 | ND |
| Heng fang | 3.5×10^5^ | 5.7×10^3^ | 0 | 0 | 2.4×10^2^ | 0 | 0 | ND |
| Loreal | 2.6×10^4^ | 3.0×10^3^ | 0 | 0 | 6.7×10^1^ | 0 | 0 | ND |
| **Mehedi**  (Akon *et al*., 2015) |  |  |  |  |  |  |  |  |
| Mumtaz gold no-1 | 1.3×10^5^ | 3.1×10^4^ | 0 | 0 | 2.0×10^2^ | 4.0×10^2^ | 0 | ND |
| Ligion active gold | 2.0×10^4^ | 4.1×10^3^ | 0 | 0 | 3.1×10^2^ | 2.4×10^2^ | 0 | ND |
| Smart active | 1.40×10^5^ | 4.0×10^2^ | 0 | 0 | 0 | 0 | 0 | ND |
| **Hair remover cream**  (Akon *et al*., 2015) |  |  |  |  |  |  |  |  |
| Nair hair remover | 1.5×10^4^ | 2.5×10^4^ | 0 | 0 | 0 | 0 | 0 | ND |

Continued to next page

| Fem anti darkening | 2.0×10^5^ | 2.0×10^4^ | 0 | 0 | 0 | 2.0×10^1^ | 0 | ND |
| --- | --- | --- | --- | --- | --- | --- | --- | --- |
| Cosmo silky | 3.1×10^5^ | 3.1×10^4^ | 0 | 1.5×10^5^ | 3.0×10^3^ | 4.0×10^2^ | 0 | ND |
| **Hair gel**  (Akon *et al*., 2015) |  |  |  |  |  |  |  |  |
| Extrme style | 1.5×10^5^ | 7.0×10^3^ | 0 | 0 | 2.0×10^2^ | 2.0×10^2^ | 0 | ND |
| Set in style | 1.0×10^5^ | 2.0×10^2^ | 0 | 0 | 1.5×10^3^ | 3.0×10^1^ | 0 | ND |
| Pop popular | 2.0×10^5^ | 1.0×10^3^ | 0 | 0 | 3.0×10^2^ | 2.0×10^2^ | 0 | ND |
| Gillette | 3.1×10^4^ | 1.1×10^1^ | 0 | 0 | 2.1×10^1^ | 3.0×10^2^ | 0 | ND |
| **Sunscreen Lotion**  (Akon *et al*., 2015) |  |  |  |  |  |  |  |  |
| Neutrogena | 1.0×10^5^ | 2.0×10^2^ | 0 | 0 | 1.0×10^2^ | 1.5×10^1^ | 0 | ND |
| Loreal Paris | 1.0×10^5^ | 3.0×10^3^ | 0 | 0 | 2.0×10^3^ | 1.4×10^1^ | 0 | ND |
| SomisAyurvedic | 1.5×10^5^ | 2.0×10^3^ | 0 | 0 | 1.4×10^2^ | 2.0×10^2^ | 0 | ND |
| Boro Plus | 1.3×10^5^ | 4.0×10^3^ | 0 | 0 | 3.0×10^3^ | 1.2×10^1^ | 0 | ND |
| **Lip gloss**  ( Akon et al., 2015) |  |  |  |  |  |  |  |  |
| Ludanmei | 1.5×10^5^ | 2.0×10^3^ | 0 | 0 | 2,5×10^3^ | 2.0×10^2^ | 0 | ND |
| Drevn | 1.1×10^5^ | 1.0×10^2^ | 0 | o | 3.0×10^2^ | 2.0×10^1^ | 0 | ND |
| Clipon | 1.2×10^5^ | 5.0×10^3^ | 0 | 0 | 2.0×10^3^ | 3.0×10^1^ | 0 | ND |
| **Moisturizer Cream**  (Akon *et al*., 2015) |  |  |  |  |  |  |  |  |
| Clean & Clear | 4.0×10^5^ | 1-2×10^3^ | 0 | 1.0×10^3^ | 1.4×10^3^ | 1.3×10^2^ | 0 | ND |
| Olay | 3.0×10^5^ | 1.0×10^2^ | 0 | 0 | 1.0×10^3^ | 1.0×10^1^ | 0 | ND |
| **Antiageing Cream**  (Akon *et al*., 2015) |  |  |  |  |  |  |  |  |
| Pond’s | 3.0×10^5^ | 2.0×10^2^ | 0 | 2.0×10^3^ | 2.5×10^3^ | 2.0×10^2^ | 0 | ND |
| Olay | 4.0×10^5^ | 1.0×10^2^ | 0 | 0 | 0 | 1.0×10^1^ | 0 | ND |
| ***Soap*** |  |  |  |  |  |  |  |  |
| Lux | 2.3×10^5^ | 2.1×10^3^ | 0 | 5.4×10^1^ | 2.6×10^1^ | 1.6×10^1^ | 0 | 0 |
| Dettol | 2.1×10^3^ | 3.2×10^2^ | 0 | 4.6×10^1^ | 7.6×10^1^ | 0 | 0 | 0 |
| Wheel  (Detergent) | 2.2×10^4^ | 4.5×10^2^ | 0 | 5.6×10^1^ | 1.6×10^1^ | 6.1×10^1^ | 0 | 0 |

Continued to next page

| ***Shampoo***  (Das *et al*., 2013) |  |  |  |  |  |  |  |  |
| --- | --- | --- | --- | --- | --- | --- | --- | --- |
| Dove | 1.1×10^3^ | 7.7×10^2^ | 0 | 2.4×10^1^ | 6.2×10^1^ | 0 | 0 | 0 |
| Head & Shoulder | 2.8×10^4^ | 2.5×10^2^ | 0 | 0 | 1.0×10^2^ | 0 | 0 | 0 |
| Sunsilk | 1.8×10^5^ | 2.2×10^2^ | 0 | 1.8×10^1^ | 7.2×10^2^ | 3.3×10^1^ | 0 | 0 |
| All Clear | 7.4×10^3^ | 7.2×10^2^ | 0 | 1.6×10^1^ | 9.68×10^2^ | 1.5×10^1^ | 0 | 0 |
| ***Body lotion***  (Das *et al*., 2013) |  |  |  |  |  |  |  |  |
| Johnson | 1.8×10^3^ | 5.0×10^2^ | 0 | 1.5×10^1^ | 2.7×10^1^ | 1.2×10^1^ | 0 | 1.0×10^1^ |
| Meril | 5.5×10^5^ | 5.7×10^2^ | 0 | 0 | 1.4×10^1^ | 2.5×10^2^ | 0 | 5.5×10^1^ |
| Nevia | 2.6×10^3^ | 5.1×10^1^ | 0 | 1.5×10^1^ | 6.7×10^1^ | 3.2×10^1^ | 0 | 2.0×10^1^ |
| Ponds | 1.9×10^5^ | 3.5×10^2^ | 0 | 2.5×10^1^ | 2.7×10^2^ | 7.9×10^2^ | 0 | 5.3×10^1^ |
| ***Face wash***  (Das *et al*., 2013) |  |  |  |  |  |  |  |  |
| Garnier | 6.91×10^3^ | 2.50×10^2^ | 0 | 0 | 0 | 0 | 0 | 2.5×10^1^ |
| Johnson | 1.10×10^4^ | 4.69×10^2^ | 0 | 0 | 2.56×10^2^ | 2.96×10^2^ | 0 | 2.00×10^2^ |
| Ponds | 1.40×10^5^ | 1.06×10^2^ | 0 | 2.00×10^1^ | 5.14×10^2^ | 8.26×10^2^ | 0 | 5.00×10^2^ |
| ***Cream***  (Das *et al*., 2013) |  |  |  |  |  |  |  |  |
| Fair & Lovely | 5.95×10^5^ | 0 | 0 | 5.00×10^1^ | 5.20×10^2^ | 1.50×10^2^ | 0 | 4.50×10^2^ |
| Johnson | 8.28×10^3^ | 0 |  | 1.50×10^1^ | 2.00×10^1^ | 1.65×10^1^ | 0 | 3.00×10^2^ |
| Ponds | 8.94×10^4^ | 2.24×10^2^ | 0 | 1.29×10^1^ | 5.30×10^2^ | 0 | 0 | 2.00×10^2^ |
| ***Petroleum***  (Das *et al*., 2013) |  |  |  |  |  |  |  |  |
| Vaseline | 1.5×10^3^ | 3.0×10^1^ | 0 | 0 | 1.8×10^2^ | 0 | 0 | 0 |
| Meril | 1.3×10^5^ | 1.5×10^2^ | 0 | 0 | 2.5×10^2^ | 1.2×10^2^ | 0 | 5.5×10^2^ |
| Tibet | 1.2×10^4^ | 1.6×10^2^ | 0 | 0 | 5.4×10^3^ | 3.0×10^1^ | 0 | 0 |

ND - Not Done. TVB Total Viable Bacteria

TVBC Total viable bacterial count

USP or FDA limit of aerobic bacteria <10^3^ cfu g^-1^ for products of non-eye area

Continued to next page

As has already been described in the published papers cited, all samples were aseptically collected from the different healthcare stationary shops in Dhaka city, homogenized with sterile peptone buffer water (pH 7.2 ± 0.2) in 9:1 ratio, and subsequently diluted the samples up to 10^-5^ and spread on to different selective and differential media for total aerobic plate count, fungal load estimation and the quantification of the pathogenic bacteria (Akon *et al*., 2015; Das *et al*., 2013; Cappuccino & Sherman, 1996). The confirmative biochemical tests and the relevant statistical analyses have been performed later. All the tested samples were exhibit higher load of bacteria up to 10^5^ cfu g^-1^ which eventually exceeded the USP or FDA limit of <10^3^ cfu g^-1^ for non-eye area. In most of the cases all tested cosmetics samples were found to be contaminated with the *Staphylococcus* spp., *Pseudomonas* spp., *Klebshiella* spp. and *Bacillus* spp. On the other hand, *E. coli* and *Actinomycetes* were totally absent in all samples.
